# Case Report: Co-existence of *BCR::PDGFRA* gene fusion and *PDGFRA* variants in myeloid neoplasm with persistent leukocytosis, large splenomegaly, and eosinophilia

**DOI:** 10.3389/fonc.2026.1671293

**Published:** 2026-02-17

**Authors:** Zhifang Xiao, Chongyan Lu, Peng Zhang, Na Han, Xianjun He, Jinfeng Zhang, Yueqi Feng, Mengshan Guan, Ling Ouyang, Yang Gao, Yonghua Li

**Affiliations:** 1 Department of Hematology, General Hospital of Southern Theater Command, Guangzhou, China; 2 Guangzhou University of Chinese Medicine, Guangzhou, China

**Keywords:** *BCR::PDGFRA* fusion, imatinib, myeloproliferative neoplasm, *PDGFRA* variants, tyrosine kinase inhibitors

## Abstract

Persistent leukocytosis, massive splenomegaly, and eosinophilia are common manifestations in patients with myeloproliferative neoplasms (MPNs), particularly in those with chronic myeloid leukemia (CML). CML is characterized by the *BCR::ABL* fusion gene, typically associated with the t(9;22)(q34;q11) translocation. Herein, we report a case of myeloid neoplasm with a rare variant translocation, t(4;22)(q12;q11), involving the *BCR::PDGFRA* fusion gene and coexisting *PDGFRA* variants, accompanied by persistent leukocytosis, massive splenomegaly, and eosinophilia. Laboratory tests showed elevated white blood cell counts, with increased monocytes, neutrophils, and eosinophils. Bone marrow aspiration revealed a granulocytic-erythrocytic ratio of 189:1, marked granulocytic hyperplasia, and numerous immature granulocytes. Genetic testing confirmed an uncommon *BCR::PDGFRA* and coexisting *PDGFRA* mutations (c.1666G>A and c.1701A>G), confirming the diagnosis of myeloid neoplasm with *BCR::PDGFRA* rearrangement. Treatment with imatinib, a tyrosine kinase inhibitor, resulted in a continuous complete molecular response (CMR). To our knowledge, this is the first report to demonstrate the clinical and cytogenetic manifestations of *BCR::PDGFRA* positive myeloid neoplasm coexisting *PDGFRA* mutations. Furthermore, it emphasizes the effectiveness of targeted therapy and the significance of personalized management.

## Introduction

Myeloid neoplasms, characterized by abnormal proliferation and accumulation of myeloid cells, require an integrated diagnostic approach encompassing clinical presentation, peripheral blood analysis, bone marrow examination, and cytogenetic/molecular testing to prevent misdiagnosis (1). *The breakpoint cluster region (BCR)* gene, located on chromosome 22q11, fused with *the Abelson murine leukemia (ABL)* gene on chromosome 9q34, is the hallmark of chronic myelogenous leukemia (CML) (2, 3). Previous reports indicate that *BCR* cytogenetic variants do not always involve *BCR::ABL* fusion gene (two separate genes abnormally join together, forming a novel fusion gene); instead, *BCR* has been shown to fuse with other tyrosine kinase genes, including *fibroblast growth factor receptor 1 (FGFR1)*, *platelet-derived growth factor receptor alpha (PDGFRA)*, *ret proto-oncogene (RET)*, and *JAK2 (*4). The most common *BCR* fusion is *BCR::ABL*, while *BCR::PDGFRA* is rare. *BCR::PDGFRA* was first identified in patients with atypical chronic myeloid leukemia (aCML) associated with the t(4;22)(q12;q11) translocation (5). This disorder type is classified under Myeloid/Lymphoid neoplasms with eosinophilia and tyrosine kinase gene fusions (MLN-TK), a new entity defined in the WHO 2022 Classification of Hematolymphoid Tumors and is labeled as sensitive to tyrosine kinase inhibitors (TKIs, a class of targeted drugs that block the activity of abnormal proteins driving cancer growth) (6, 7). Otherwise, an activating mutation of *PDGFRA* is also sensitive to TKIs. Therefore, identifying rare fusion genes like *BCR::PDGFRA* or *PDGFRA* mutations is critical because they often predict responsiveness to targeted therapies, offering patients more effective and less toxic treatment options compared to conventional chemotherapy. However, co-existence of *BCR::PDGFRA* and *PDGFRA* variants in one patient has not been reported and the sensitivity to TKIs is unknown.

## Case presentation

**Figure 1:**
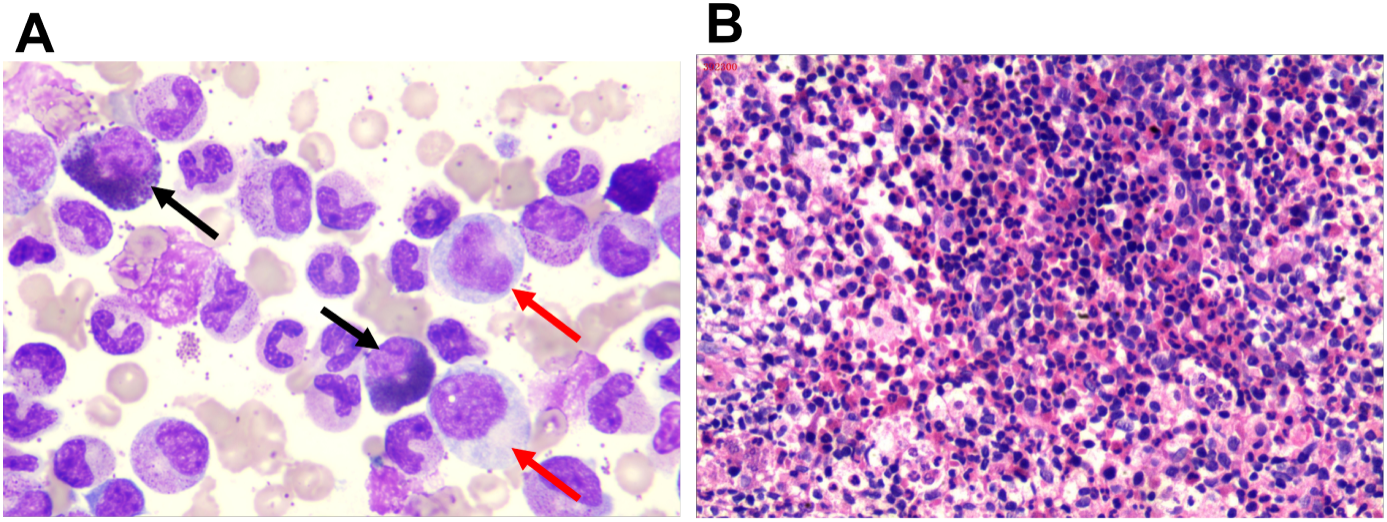
Bone marrow morphological characteristics of the patient. **(A)** Bone marrow smear showing granulocytic hyperplasia. Red arrows point to nuclear-cytoplasmic asynchrony, and the black arrow indicates eosinophilia (10×100). **(B)** Bone marrow biopsy stained with hematoxylin and eosin, revealing a hypercellular marrow with an increased granulocyte to erythrocyte ratio.

A 59-year-old man visited the hospital on June 06, 2023, after complaining of abdominal distension and fatigue for one month. The physical examination revealed abdominal bloating and an enlarged spleen. There was no significant swelling of the superficial lymph nodes or liver. Blood tests revealed significant leukocytosis, with an elevated white blood cell (WBC) count of 152.61×10^9^/L. Neutrophils accounted for 84% of the WBC, eosinophils for 4%, and monocytes for 9%. Low hemoglobin levels (57 g/L) and reduced platelet count (63×10^9^/L). The peripheral blood smear revealed an increase in neutrophil precursors, with myelocytes at 9% and metamyelocytes at 5%. Bone marrow aspiration revealed hypercellularity, with a granulocytic to erythrocytic ratio (G/E ratio) of 189:1 and granulocytic hyperplasia accounting for 94.5% of cells. Twenty percent of the neutrophilic myelocytes showed megaloblastoid alterations and occasional nuclear-cytoplasmic asynchrony. The eosinophil count was increased, accounting for 9% of all cells with normal shape (**Figure 1A**). An examination of the entire slide indicated erythroid, lymphoid, and megakaryocytic cells all had normal morphology, with no dysplastic characteristics. Bone marrow biopsy revealed a very vigorous growth of hematopoietic tissue with essentially no adipose tissue (**Figure 1B**). The hematopoietic tissue had a considerably higher G/E ratio, with numerous segmented neutrophils and a few immature myeloid cells. The abdominal ultrasonography revealed large splenomegaly with a longitudinal dimension of 17.51 cm, thickness of 6.23 cm, and 5.4 cm extension below the ribcage.

These findings strongly suggest a myeloid neoplasm. However, tests for the *BCR::ABL* (P210, P190, and P230) were negative. Additionally, mutations in *JAK2V617F*, exon 9 of the *CALR* gene, and MPL w515L/K were also negative. Morphologically, the findings support the diagnosis of atypical chronic myeloid leukemia (aCML) with *BCR::ABL* negative. Fluorescence *in situ* hybridization (FISH) was performed to detect the *FIP1L1::PDGFRA* because of obvious hypereosinophils. The chromosomal regions 4q12 of the *FIP1LI* gene locus, *CHIC2*, and *PDGFRA* were labeled with green, red, and cyan respectively. According to the literature on FISH detection of *CHIC2* deficiency, *FIP1L1::PDGFRA* fusion, and *PDGFRA* translocation (8), it is known that if there is *FIP1L1::PDGFRA*, it appears as a dual color fusion signal of green and cyan. Obviously, our FISH testing did not detect this signal.

**Figure 2:**
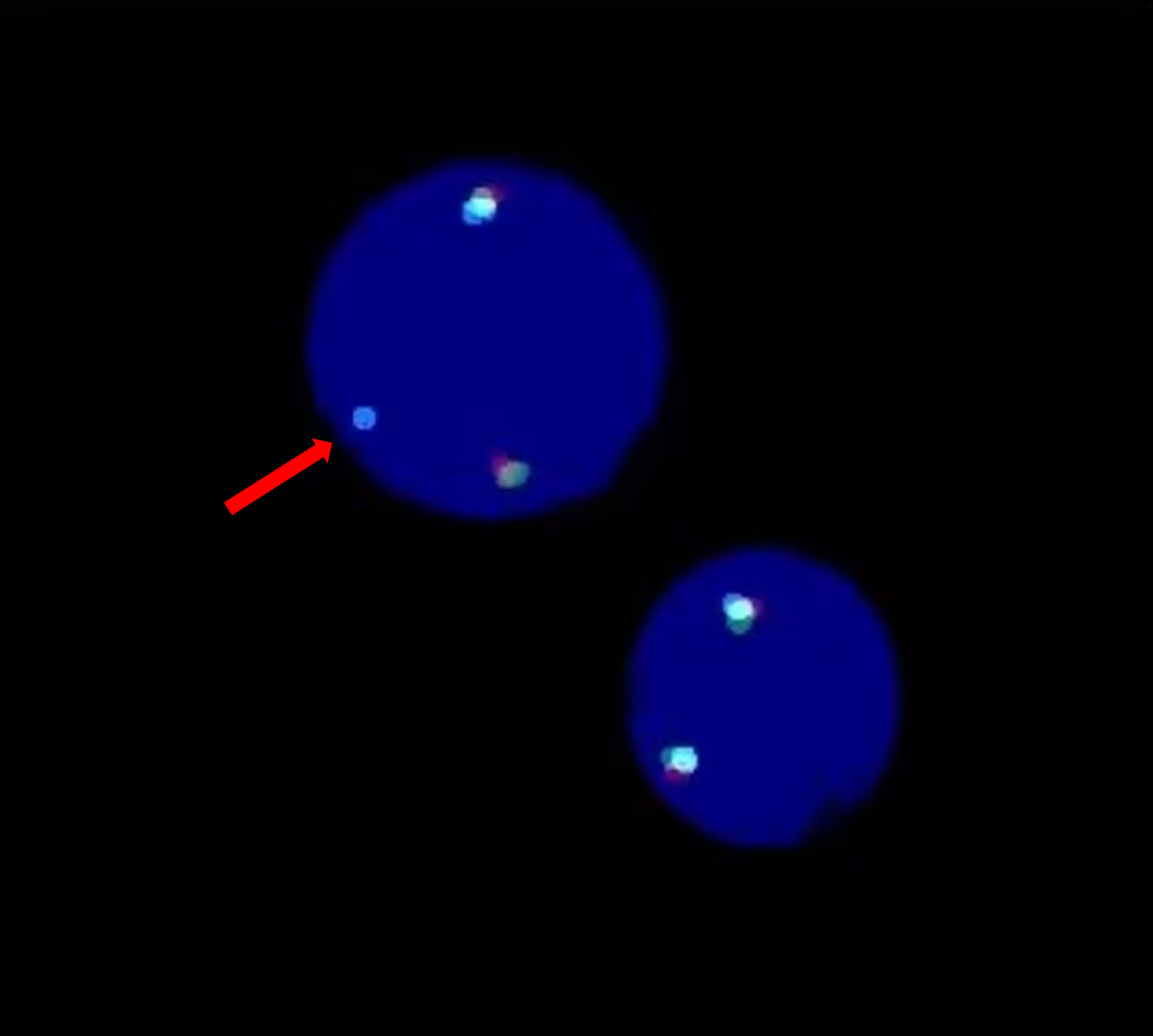
FISH image showing a rearrangement of the *PDGFRA* gene. A separate signal for *PDGFRA* (cyan) was detected (red arrow).

However, 64% of cells exhibited a separate signal for *PDGFRA* (cyan), distinct from the green and red signals, indicating a rearrangement of *PDGFRA* (**Figure 2**).

**Figure 3:**
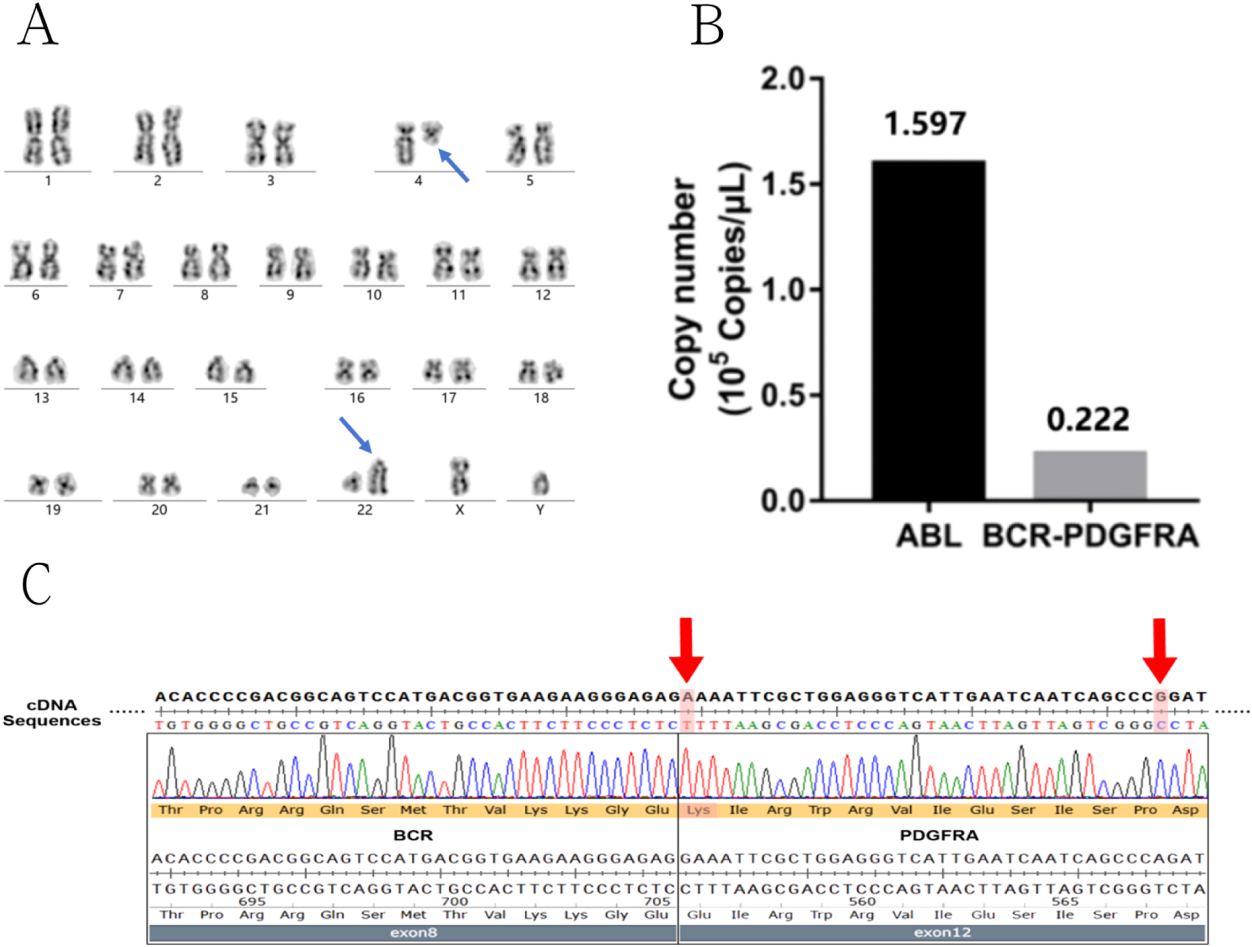
Genetic detection results of *BCR::PDGFRA* fusion and related mutations in the patient. **(A)** Karyotype analysis displaying t(4;22) translocation (blue arrow). **(B)** Quantitative real-time PCR analysis of *BCR::PDGFRA* gene showed positive fusion copies of 13.91%. **(C)** Sequencing analysis the fusion breakpoint was located at the junction of exon 8/9 of the *BCR* and exon 12 of the *PDGFRA*. The two mutations on the exon12 of *PDGFRA* were also detected (red arrow).

Chromosomal karyotype analysis revealed 46,XY,t(4;22)(q12;q11), implying that the companion gene for the rearrangement could be the *BCR* gene on chromosome 22q11 (**Figure 3A**). Real-time reverse transcription polymerase chain reaction (real-time RT-PCR) validated the fusion of the *BCR::PDGFRA* (**Figure 3B**). The fusion breakpoint was found at the junction of exons 8/9 of the *BCR* and exon 12 of the *PDGFRA*, according to genetic sequencing. Furthermore, two mutations were found on the cDNA of the *BCR::PDGFRA*, within the *PDGFRA* gene (reference transcript: NM_006206.6) on exon 12: (1) a missense mutation, c.1666G>A, p.Glu556Lys, and (2) a synonymous mutation, c.1701A>G, p.Pro567= (**Figure 3C**). The c.1666G>A is novel and has not been descripted in the gnomAD data (https://gnomad.broadinstitute.org/). Through multi-dimensional analysis using SIFT, PolyPhen-2, CADD, and ClinPred scores, it was discovered that missense mutation c.1666G>A has pathogenicity. This indicates that the presence of c.1666G>A mutation probably further aggravates the occurrence and development of the disease. As for c.1701A>G mutation, it is described as benign in the gnomAD data. At the same time, using the splicing prediction tool SpliceAI, it was found that synonymous mutation c.1701A>G does not disrupt canonical or cryptic splice sites and is non-pathogenic. The c.1701A>G mutation has no clinical significance. Hence, the patient was diagnosed with myeloid neoplasms including the *BCR::PDGFRA* and coexisting *PDGFRA* mutations.

The *BCR::PDGFRA* induces gain-of-function modifications in *PDGFRA*, which are sensitive to TKIs like imatinib and facilitating the reduction of leukemic cell proliferation and improving patient outcomes (4). Based on previous reports *PDGFRA* rearrangement is relatively sensitive to imatinib. Therefore, the patient was initially treated with hydroxyurea to reduce WBC. On June 20, 2023, he initiated imatinib (100 mg/day) as an oral tyrosine kinase inhibitor, following a reduction in WBC count to 50×10^9^/L. Three months after treatment, WBC numbers normalized, anemia improved to 122 g/L, and platelet count recovered to 102×10^9^/L, resulting in hematological remission. After six months of treatment, the spleen size was significantly reduced. The *BCR::PDGFRA* was no longer detectable by the quantitative RT-PCR test, indicating a complete molecular response (CMR, defined as the absence of detectable disease-specific molecular markers). The patient’s condition has stabilized as of Dec 2025, and he is being monitored further.

## Discussion

Importantly, it is understood that the most common rearrangement involving the *PDGFRA* gene is the *FIP1L1::PDGFRA* (9).Several fusion partners of *PDGFRA* in MPNs associated with eosinophilia, including *BCR, ETV6, KIF5B, CDK5RAP2, STRN, TNKS2*, and *FOXP1*, have been documented in the form of case reports/series (5, 10–15). The Philadelphia chromosome was not found in our report. Alternatively, we discovered that the *BCR::PDGFRA* was positive. *PDGFRA* gene on the chromosome 4q12 region encodes a tyrosine kinase receptor (16). According to the literature, five other *PDGFRA* fusion partners and more than 20 *PDGFRB* fusion partners have been reported in association with eosinophilia-associated myeloproliferative neoplasms. Although these abnormalities are very uncommon, they are associated with excellent responses to imatinib (17–19). Furthermore, our case meets the diagnostic criteria of MLN-TK as it carries the *BCR::PDGFRA* and presents with eosinophilia (6). Therefore, a tyrosine kinase inhibitor, imatinib, is the first line therapeutic agent for the treatment of this category of diseases. In this example, Imatinib 100mg once daily was administered as a first-line treatment when white blood cells decreased below 50 ×10^9^/L by utilizing hydroxyurea. Imatinib particularly inhibits the tyrosine kinase activity of the fusion protein, leading to a reduction in leukemic cell growth. The dose and length of imatinib or other TKIs therapy are dictated by the patient’s reaction and tolerance to the medicine. Regular follow-up with complete blood counts, bone marrow exams, and molecular testing is required to assess treatment response and detect any evidence of disease progression or transformation to acute leukemia. Treatment is highly customized and is determined by a lot of characters, including the patient’s age, overall health, illness features, and therapy response. To get the best potential outcome, myeloid neoplasms with *BCR::PDGFRA* should be managed by a team of hematology and oncology specialists.

**Table 1** shows that only 12 cases of *BCR::PDGFRA* (including our present case) have been recorded worldwide up to now. The review included cases of myeloid neoplasm (n=1), CML (n=1), atypical CML (n=2), CML-like myeloproliferative disorder (n=1), chronic eosinophilic leukemia (CEL, n=2), therapy-related AML (n=1), mixed phenotypic acute leukemia (n=1), T-lymphoblastic leukemia/lymphoma (T-ALL, n=1), Pre-B cell ALL (n=1), and myeloproliferative. These studies demonstrate the diversity of hematopoietic malignancies resulting from *BCR::PDGFRA*, as well as the critical necessity for precise diagnostic tools to guide proper treatment. All patients (6/6, including our present case) who received imatinib treatment survived, and the others treated with chemotherapy or stem cell transplantation had a worse survival outcome. The findings further demonstrate the efficacy of targeted therapy. As for now, these cases have been classified into myeloid/lymphoid neoplasms with *PDGFRA* rearrangement.

**Table 1:** Clinical presentations and treatment outcomes of cases with *BCR::PDGFRA*.

Case	Age/sex	Clinical assessment	CBC	Peripheral blood smear	Bone marrow examination	Diagnosis	Treatment	Outcomes
1(the present case)	59/M	Bilateral lower limb edema; splenomegaly	Increased platelet count, neutrophil number, leukocyte, and erythrocyte.	Immature myeloid cells	hypercellular	Myeloid neoplasm	Imatinib	Survival
2 (21)	32/M	Splenomegaly	Leukocytosis	Immature granulocytes, basophilia, and eosinophilia	hypercellular	CML	Imatinib	Survival
3 (5)	37/M	Splenomegaly	Leukocytosis	Increase of eosinophils and also of normal and atypical megakaryocytes.	Myeloid hyperplasia with pronounced promyelocytes and some blasts	Atypical CML	Matched alloHSCT	Survival
4 (11)	57/M	Splenomegaly Lymphadenopathy	Leukocytosis	Anemia, leukocytosis with increased granulocytes and precursors	Myeloid hyperplasia and mild eosinophilia	Atypical CML	Imatinib	Survival
5 (5)	3/M	Enlarged tonsils; lymphadenopathy liver and spleen enlargement	Leukocytosis	NA	myeloid hyperplasia	CML-like myeloproliferative disorder with extramedullary T-lymphoid blast crisis	Auto-HSCT PR Allo-HSCT (MSD)	Dead
6 (22)	37/M	Abdominal discomfort and palpable spleen	Leukocytosis	NA	NA	Myeloproliferative neoplasm	Imatinib	Survival
7 (10)	47/M	Diffuse ecchymosis Multiple lymphadenopathies Hepatosplenomegaly	Leukocytosis	NA	Increased cellularity with 73% blast cells	Pre-B cell ALL	Imatinib	Survival
8 (12)	37/M	NA	NA	NA	NA	CEL	NA	NA
9 (12)	41/M	NA	NA	NA	NA	CEL	NA	NA
10 (13)	45/W	Cervical lymphadenopathy	Leukocytosis	A predominance of blasts, medium to large with oval or irregular nuclei, condensed chromatin, negligible nucleoli, and minimal, ungranulated cytoplasm without Auer rods.	Predominantly composed of blasts	Mixed phenotypic acute leukemia	Imatinib mesylate, cytarabine, and idarubicin	Survival
11 (14)	56/M	Splenomegaly and lymphadenopathy	Leukocytosis	NA	Immature lymphoid cells	T-ALL	Induction CR within 3mo after the diagnosis; intensive induction and consolidation Regimens, treatment was followed by maintenance therapy for a total of 2 year	Survival
12 (23)	77/M	Lymphadenopathy	Leukocytosis	Increased white cell count with rapidly elevated eosinophilia and basophilia	Multilineage dysplasia and focal eosinophilia.	Therapy related AML	Intensive chemotherapy received for B-ALL and complex karyotype	Dead

Genetic testing is critical for identifying specific gene mutations associated with myeloid neoplasms, such as *JAK2, CALR*, and *MPL* (20). In this case, the identification of two mutations in exon 12 of the *PDGFRA* gene—the missense mutation c.1666G>A, which results in the amino acid substitution p.Glu556Lys, and the synonymous mutation c.1701A>G, which does not change the amino acid p.Pro567—adds another layer of complexity to the patient’s genetic profile and potential pathogenesis. To further verify the significance of genetic mutations, the SIFT, PolyPhen-2, CADD, ClinPred scores and Splice AI prediction technology provided sufficient evidence. The c.1666G>A missense mutation is pathogenic. It remains unclear whether this mutation is a gain-of-function mutation or a loss-of-function mutation. There are no relevant studies at present, and future research is required to elucidate the correlation between this mutation and the manifestation of the disease. Furthermore, multi-dimensional technical detection proves that synonymous c.1701A>G mutations do not affect the function of the gene.

Anyway, through targeted therapy (imatinib), the patient has been well controlled as well as other MLN-TK with *PDGFRA* rearrangement (**Table 1**). Our case study has confirmed that even in patients with *PDGFRA* mutations, imatinib can achieve CMR for those with *BCR::PDGFRA*. This avoids unnecessary chemotherapy for these patients and ensures that they receive the most appropriate targeted therapy.

Furthermore, this brief communication adds to the literature on this rare entity, highlighting the importance of targeted therapy in Ph negative myeloproliferative neoplasms.

## Key takeaways

The *BCR::PDGFRA* has only been reported in 11 cases in the literature. Our case discovery revealed that the patient not only had *BCR::PDGFRA* but also *PDGFRA* mutations. Moreover, the *PDGFRA* c.1666G>A mutation is novel and its coexistence with *PDGFRA* rearrangement is the first report up to now. Although the effect of c.1666G>A mutation in the *BCR::PDGFRA* is unknown, the targeted therapy using imatinib can enable the patient to achieve CMR. This indicates that timely targeted treatment can have a positive effect on such patients.

## Data Availability

The datasets presented in this study can be found in online repositories. The names of the repository/repositories and accession number(s) can be found in the article/supplementary material.

## References

[1] Thapa B Fazal S Parsi M Rogers HJ . Myeloproliferative neoplasms. In: Statpearls. Treasure Island (FL): StatPearls Publishing (2025). Available online at: http://www.ncbi.nlm.nih.gov/books/NBK531464/. 30285359

[2] Suryanarayan K Hunger SP Kohler S Carroll AJ Crist W Link MP . Consistent involvement of the BCR gene by 9;22 breakpoints in pediatric acute leukemias. Blood. (1991) 77:324–30. doi: 10.1182/blood.V77.2.324.324, PMID: 1985699

[3] Roumiantsev S Krause DS Neumann CA Dimitri CA Asiedu F Cross NC . Distinct stem cell myeloproliferative/T lymphoma syndromes induced by ZNF198-FGFR1 and BCR-FGFR1 fusion genes from 8p11 translocations. Cancer Cell. (2004) 5:287–98. doi: 10.1016/s1535-6108(04)00053-4, PMID: 15050920

[4] Peiris MN Li F Donoghue DJ . BCR: A promiscuous fusion partner in hematopoietic disorders. Oncotarget. (2019) 10:2738–2754. doi: 10.18632/oncotarget.26837, PMID: 31105873 PMC6505627

[5] Baxter EJ Hochhaus A Bolufer P Reiter A Fernandez JM Senent L . The t(4;22)(q12;q11) in atypical chronic myeloid leukaemia fuses BCR to PDGFRA. Hum Mol Genet. (2002) 11:1391–7. doi: 10.1093/hmg/11.12.1391, PMID: 12023981

[6] Reiter A Metzgeroth G Cross NCP . How I diagnose and treat myeloid/lymphoid neoplasms with tyrosine kinase gene fusions. Blood. (2025) 145:1758–68. doi: 10.1182/blood.2023022417, PMID: 39046810

[7] Kaur P Khan WA . Myeloid/lymphoid neoplasms with eosinophilia and platelet derived growth factor receptor alpha (PDGFRA) rearrangement. In: Li W , editor. Leukemia. Brisbane (AU): Exon Publications (2022). Available online at: http://www.ncbi.nlm.nih.gov/books/NBK586201/., PMID: 36395310

[8] Fink SR Belongie KJ Paternoster SF Smoley SA Pardanani AD Tefferi A . Validation of a new three-color fluorescence in *situ* hybridization (FISH) method to detect CHIC2 deletion, FIP1L1/PDGFRA fusion and PDGFRA translocations. Leuk Res. (2009) 33:843–6. doi: 10.1016/j.leukres.2008.11.016, PMID: 19118897

[9] Cools J DeAngelo DJ Gotlib J Stover EH Legare RD Cortes J . A tyrosine kinase created by fusion of the PDGFRA and FIP1L1 genes as a therapeutic target of imatinib in idiopathic hypereosinophilic syndrome. N Engl J Med. (2003) 348:1201–14. doi: 10.1056/NEJMoa025217, PMID: 12660384

[10] Reiter A Gotlib J . Myeloid neoplasms with eosinophilia. Blood. (2017) 129:704–714. doi: 10.1182/blood-2016-10-695973, PMID: 28028030

[11] Trempat P Villalva C Laurent G Armstrong F Delsol G Dastugue N . Chronic myeloproliferative disorders with rearrangement of the platelet-derived growth factor alpha receptor: A new clinical target for STI571/glivec. Oncogene. (2003) 22:5702–6. doi: 10.1038/sj.onc.1206543, PMID: 12944919

[12] Safley AM Sebastian S Collins TS Tirado CA Stenzel TT Gong JZ . Molecular and cytogenetic characterization of a novel translocation t(4;22) involving the breakpoint cluster region and platelet-derived growth factor receptor-alpha genes in a patient with atypical chronic myeloid leukemia. Genes Chromosomes Cancer. (2004) 40:44–50. doi: 10.1002/gcc.20014, PMID: 15034867

[13] Erben P Gosenca D Müller MC Reinhard J Score J Del Valle F . Screening for diverse PDGFRA or PDGFRB fusion genes is facilitated by generic quantitative reverse transcriptase polymerase chain reaction analysis. Haematologica. (2010) 95:738–44. doi: 10.3324/haematol.2009.016345, PMID: 20107158 PMC2864379

[14] Wang HY Thorson JA Broome HE Rashidi HH Curtin PT Dell’Aquila ML . t(4;22)(q12;q11.2) involving presumptive platelet-derived growth factor receptor a and break cluster region in a patient with mixed phenotype acute leukemia. Hum Pathol. (2011) 42:2029–36. doi: 10.1016/j.humpath.2010.07.028, PMID: 21676437

[15] Yigit N Wu WW Subramaniyam S Mathew S Geyer JT . BCR-PDGFRA fusion in a T lymphoblastic leukemia/lymphoma. Cancer Genet. (2015) 208:404–7. doi: 10.1016/j.cancergen.2015.04.007, PMID: 26095243

[16] Curtis CE Grand FH Musto P Clark A Murphy J Perla G . Two novel imatinib-responsive PDGFRA fusion genes in chronic eosinophilic leukaemia. Br J Haematol. (2007) 138:77–81. doi: 10.1111/j.1365-2141.2007.06628.x, PMID: 17555450

[17] Jovanovic JV Score J Waghorn K Cilloni D Gottardi E Metzgeroth G . Low-dose imatinib mesylate leads to rapid induction of major molecular responses and achievement of complete molecular remission in FIP1L1-PDGFRA-positive chronic eosinophilic leukemia. Blood (2007) 109:4635–40. doi: 10.1182/blood-2006-10-050054, PMID: 17299092

[18] David M Cross NC Burgstaller S Chase A Curtis C Dang R . Durable responses to imatinib in patients with PDGFRB fusion gene-positive and BCR-ABL-negative chronic myeloproliferative disorders. Blood. (2007) 109:61–4. doi: 10.1182/blood-2006-05-024828, PMID: 16960151

[19] Apperley JF Gardembas M Melo JV Russell-Jones R Bain BJ Baxter EJ . Response to imatinib mesylate in patients with chronic myeloproliferative diseases with rearrangements of the platelet-derived growth factor receptor beta. N Engl J Med. (2002) 347:481–7. doi: 10.1056/NEJMoa020150, PMID: 12181402

[20] Easwar A Siddon AJ . Genetic landscape of myeloproliferative neoplasms with an emphasis on molecular diagnostic laboratory testing. Life (Basel) (2021) 11:1158. doi: 10.3390/life11111158, PMID: 34833034 PMC8625510

[21] Gao L Xu Y Weng LC Tian ZG . A rare cause of persistent leukocytosis with massive splenomegaly: Myeloid neoplasm with BCR-PDGFRA rearrangement-case report and literature review. Med (Baltimore). (2022) 101:e29179. doi: 10.1097/MD.0000000000029179, PMID: 35713428 PMC9276081

[22] Singh MK Sasikumaran Nair Remani A Bhave SJ Mishra DK Arora N Parihar M . Detection of BCR/PDGRFα fusion using dual colour dual fusion BCR/ABL1 probe: an illustrative report. Indian J Hematol Blood Transfus. (2019) 35:570–574., PMID: 31388278 10.1007/s12288-019-01095-9 PMC6646510

[23] Zhou J Papenhausen P Shao H . Therapy-related acute myeloid leukemia with eosinophilia, basophilia, t(4;14)(q12;q24) and PDGFRA rearrangement: A case report and review of the literature. Int J Clin Exp Path. (2015) 8:5812–20., PMID: 26191303 PMC4503174

